# Primary Total Knee Replacement: A Recipe for Dry Dressing

**DOI:** 10.7759/cureus.37820

**Published:** 2023-04-19

**Authors:** Muhammad Azfar Khanzada, Omer Adil Awadelkarim, Tarig Abdel Rahman, Adel Ghandour

**Affiliations:** 1 Orthopedics, Dr. Sulaiman Al Habib Suwaidi Hospital, Riyadh, SAU; 2 Orthopedics/Anesthesiology, Dr. Sulaiman Al Habib Suwaidi Hospital, Riyadh, SAU

**Keywords:** total knee replacement (tkr), surgical outcome, perioperative care, osteoarthritis, carboxymethylcellulose sodium

## Abstract

Objective: To evaluate surgical outcomes using sets of techniques to achieve single dry dressing for two weeks post total knee replacement (TKR) at Dr. Sulaiman Al-Habib Hospital, Riyadh, Saudi Arabia.

Material and methods: A prospective study comprised of 110 consecutive unilateral total knee replacements was conducted at the orthopedic department of Dr. Sulaiman Al-Habib Hospital, Suwaidi, Riyadh, KSA. Patients of both genders underwent knee replacement surgery due to primary knee osteoarthritis, Kellgren-Lawrence grades 3 and 4. Routine investigations and fitness evaluations of patients were carried out preoperatively. Preoperative minimal use of a tourniquet and release prior to arthrotomy closure; intravenous tranexamic acid, no drains; capsule infiltration with local anesthetics without adrenaline; tight closure with barbed sutures up to the skin in three layers; skin glue; Aquacel dressing; adductor canal block; and continuation of oral anticoagulant for four weeks.

Results: A total of 110 cases were included, of which 81 (73.6%) were females and 29 (26.4%) were males. The mean age of the study population was 60.5+10.3 years (48 to 88 years). The mean BMI in our patients was 30.57+10.5 kg/m^2^. Most patients were morbidly obese 13 (30.95%). The mean preoperative Hb% was 13.07+1.6 g/dl, while the mean postoperative Hb% was 12.58+1.9 mg/dl with a p-value of 0.28, which was non-significant statistically. Only two patients needed a change of Aquacel wound dressing for ooze. None of our patients developed deep venous thrombosis (DVT) or an infection.

Conclusion: A sequential use of sets of techniques is observed to be associated with improved outcomes in terms of blood loss, wound infection, mobility, and patient satisfaction, leading to the ultimate end point of dry Aquacel wound dressing.

## Introduction

Osteoarthritis (OA) is among the most common causes resulting in disability among the elderly population worldwide. It causes intense pain and functional compromise, affecting the quality of life [1]. Milder to moderate cases of OA respond well to the conservative management modalities, but the end-stage arthritis of the knee joint is a definitive indication of surgical management, thus requiring total knee arthroplasty or total knee replacement (TKR) [2].

TKR is one of the most common orthopedic operations and is the largest reconstruction market for the knee worldwide, which is increasing markedly with the passing of time. Globally, the number of TKR surgeries is expected to rise to 3.48 million by the end of 2030 [3,4]. Different factors, including aging and obesity, are the major contributing elements for arthropathies leading to knee and hip arthroplasty, which have seen a 300% increase in a decade. Several complications associated with surgical procedures, including venous thromboembolisms, wound infections, etc., are responsible for hospital readmissions [5,6].

Different strategies are developed to minimize post-TKR complications. Preoperative patient education about their self-care is one of them that improves compliance as well as postsurgical outcomes and fall injuries postoperatively [7,8]. Moreover, pre-admission testing is another strategy that minimizes post-operative complications by 60%, post-anesthesia care by 50%, and mortality by 5%. It further helps to reduce undue admissions related to other medical conditions by 38% to 66%. Prevention of deep venous thromboembolic (DVT) events is another task for TKR surgery, as it is the most common cause of cardiovascular events and re-admissions to the hospital within 90 days postoperatively that also required prophylactic use of aspirin or other anticoagulants. [9] Obesity is among the other contributory factors associated with both the intraoperative complications and the postoperative complications of joint replacement, including stroke, MI, malpositioning, hospital readmissions, revised surgery, infections, poor functional outcomes, and decreased 10-year survival [6]. Many institutes do not accept patients for knee replacement with a BMI above 40 kg/m^2^ due to their high complication rates [6,10].

Despite the advancement of surgical techniques for treating arthritis patients, the possibility of postoperative complications has also been reported worldwide. Alterations and modifications in the pre-, per-, and post-TKR techniques are other moves to improve surgical outcomes. Moreover, it may also reduce intraoperative as well as postoperative complications like bleeding, wound ooze, infection, and DVT and improve the quality of life of patients. Keeping this in view, the present study was planned with the objective to evaluate the surgical outcome amongst patients using sets of techniques to achieve single dry dressing for two weeks post total knee replacement, admitted to the orthopedic surgery department of Dr. Sulaiman Al Habib Hospital, Suwaidi, Riyadh, Saudi Arabia.

## Materials and methods

A prospective study of 110 consecutive unilateral total knee replacements was conducted at the orthopedic department of Dr. Sulaiman Al-Habib Hospital, Suwaidi, Riyadh, KSA. All the patients, of both genders, who underwent knee replacement surgery due to primary knee osteoarthritis (Kallgren Lawrence grades 3 and 4) were included in this study, while patients with secondary osteoarthritis and hypersensitivity to tranexamic acid were excluded.

Pre-operative preparation

After getting informed consent, routine investigations and the fitness of patients were carried out. The surgical procedure was carried out for all patients by the same orthopedic surgeon using the same methods and in the same sequence under general or spinal anesthesia by the hospital's expert anesthetist. Along with it, preoperative cefuroxime (1.5 g) was administered as a prophylactic measure. Moreover, a pneumatic tourniquet was applied to the upper thigh after limb elevation and exsanguination and inflated to the pressure needed in the patient accordingly.

Operative procedure

The surgical procedure was performed while keeping the patient in the supine position. A midline incision followed by a medial parapatellar arthrotomy was performed. Mono-polar electrocauterization was used to control bleeding. In all patients, cemented TK implants were used. A single intravenous dose of tranexamic acid was used during the procedure. The femoral canal was closed with a bone plug, and a trail insert was initially used for the proper size. The tourniquet was released to assess and control bleeding points at the back of the knee, and hemostasis was secured prior to inserting the proper insert. Local anesthetics without adrenaline are used for joint capsule infiltration and in the soft tissue around the knee joint. No intra-articular drain was used, and the wound was closed with barbed sutures up to the skin in three layers while skin glue was applied for the skin closure. Adductor canal block is given by the anesthesia team. An Aquacel surgical dressing was applied, followed by the compression dressing that was later removed on the first postoperative day. Physiotherapy and prophylactic oral anticoagulants (Xarelto) were commenced on postoperative day 1 as a once-daily regime for four weeks to prevent DVT. Moreover, patient education was given about the warning signs of infection and DVT on discharge, and patients were advised to visit the emergency department if needed.

Postoperative follow-up

All patients received immediate postoperative care in the recovery room, followed by care in the ward as per the hospital package of care. Hemoglobin levels are checked 12 hours postoperatively and again, if needed, within two weeks of surgery. Upon discharge, postoperative rehabilitation dates were provided to the patients as per standard protocols. On the follow-up visit, screening for DVT was arranged through Doppler ultrasound or computerized tomography (CT) angiography only in symptomatic patients. The Aquacel dressing was removed routinely on the first post-surgery visit to the clinic after two weeks, or earlier if it leaked or showed saturation of more than 50% of the inner hydrofiber layer.

Statistical analysis

Data were entered and statistically analyzed using IBM SPSS version 20.0 software (IBM Corp., Armonk, NY). A p-value of <0.05 was regarded as statistically significant when the Student’s t-test was employed as a statistical test of significance.

Ethical approval

The study was conducted after obtaining ethical clearance from the Ethics Committee of Dr. Sulaiman Al-Habib Hospital, Suwaidi, Riyadh, KSA, with the approval number REC No. HMG/SWD/REC/067.

## Results

A total of 110 cases of primary knee replacement surgery were carried out in our department, of which 81 (73.6%) were female and 29 (26.4%) were male. The mean age was 60.5+10.3 years (48 to 88 years) for our study population. The mean BMI in our patients was 30.57+10.5 kg/m^2^ (21.8 to 48.8 kg/m^2^). Table 1 presents the BMI distribution of the studied patients.

**Table 1:** BMI distribution in studied patients (n=110)

Level of BMI	n (%)
Normal	16 (14.5)
Overweight	24 (21.8)
Obesity grade 1	21 (19.1)
Obesity grade 2	16 (14.5)
Obesity grade 3	33 (30.1)

The hemoglobin level of the study patients was measured before and after knee surgery. None of the patients, whose hemoglobin level was above 11 mg/dl, needed transfusions post-surgery. The range of preoperative hemoglobin was 8.4 to 15.9, while the postoperative range was 9.5 to 14.7. Table 2 presents the mean ± SD values of pre- and post-surgical hemoglobin levels in patients as recorded for all the patients.

**Table 2:** Comparative analyses between pre and post-surgery hemoglobin levels

Parameter		Mean	P-value
Hemoglobin (mg/dl)	Before surgery	13.07±1.6	0.28
	After surgery	12.58±1.9	

**Figure 1:**
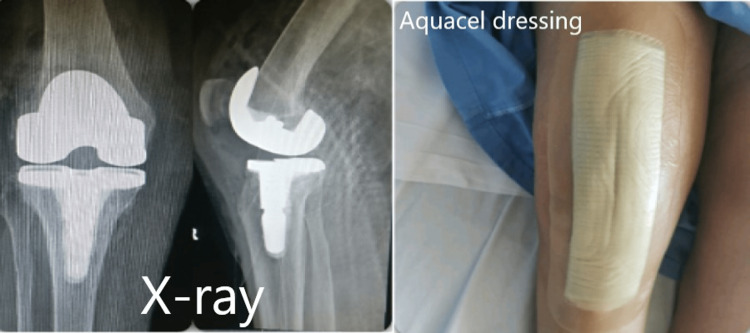
X-ray of knee replacement and Aquacel dressing

Anticoagulant was prescribed prophylactically, and Aquacel dressing was applied while no intra-articular drain was inserted in any of the patients. Only two patients needed a change of Aquacel wound dressing for ooze. None of the patients reported any postoperative wound infection, DVT, re-admission, or any other complication. Early mobility was noted in all the patients (Figure 1).

## Discussion

This modified technique showed very promising results in terms of improved outcomes, lesser blood loss, lesser chances of wound infection with early recovery, and greater satisfaction at the patient level [7]. There are more chances of blood loss, wound ooze, wound discharge, and wound infection following anticoagulants, but our modified technique and the newer oral anticoagulant showed excellent results [11]. The present study demonstrated the surgical outcome of the modified knee replacement technique. In this study, the majority of patients were female (73.6%), while the mean age of patients was 60.5+10.3 years. Our results are consistent with the results of a study by Seol et al., Zhang et al., and Lui et al., who reported that most of their patients were female and that the mean age was between 60 and 68 years [11-13]. Raised BMI is among the contributory factors allied with intra- and postoperative complications of TKR. The mean BMI in our patients was 30.57+10.5 kg/m^2^, while 30.1% of patients in this study had grade 3 obesity. Girardi et al., Keeney et al., and Feng et al. reported findings that are consistent with our study [14-16].

Tranexamic acid was given intravenously during the procedure, and it was also demonstrated that the administration of intraoperative tranexamic acid significantly improved the blood loss, reduced the transfusion rates, and prevented the postoperative drop in hemoglobin concentration in our patients. Our study demonstrated that none of the patients reported significant postoperative blood loss; these findings were also shown by Seol et al. Moreover, we checked the hemoglobin after 12 hours of TKR. They further reported that they gave the same dose of tranexamic acid (10 mg/kg body weight) twice to their patients, whereas, in the present study, we used tranexamic acid 1 g intravenously as a single dose [12]. Su et al. also reported that injecting tranexamic acid during knee replacement surgery has been a successful practice for more than a decade for the purpose of reducing blood loss, transfusion rates, and thromboembolic phenomena [17].

Wound dressings such as films, foams, hydrocolloids, alginate, antimicrobial dressings, ciNPWT, etc. are intermittently used to enhance wound management in patients after TKR. In this study, we applied Aquacel Ag dressing (silver impregnated and having antiseptic properties that sterilize the wound ooze contained) on the completion of surgery and observed a significantly improved outcome with no wound ooze or infection and early recovery, leading to the patient’s satisfaction. Moreover, only two of our patients' wound dressings need to be replaced during the postoperative period with dry wounds. Akdogan et al. and Feng et al. also used and compared Acquacel dressing with others in their studies and found consistent findings like ours [16,18]. Themistoklis et al. also suggested the application of the tourniquet and the intravenous administration of tranexamic acid during knee replacement surgery as effective measures to minimize blood loss, which is what we also recommend along with the Aquacel dressing to produce the optimized results [19].

Apixaban is a direct inhibitor of factor Xa (free as well as clot-bound); it selectively and reversibly inhibits the conversion of prothrombin into thrombin, which is the final step for the formation of a clot; it was also reported to be associated with fewer chances of bleeding postoperatively in a meta-analysis that is also in accordance with the present findings [20,21]. We prescribed Rivaroxaban 10 mg as a once-daily oral regime for four weeks for DVT prophylaxis to our patients postoperatively, which proved more convenient to the patient in comparison with apixaban, which is a twice-daily dose. Only 2 out of 110 of our patients developed wound ooze, which leads to changes in wound dressing. To the best of our knowledge, this study is one of a kind, as it was our unique effort for patient care in our current setup. There are certain limitations, like the fact that it was a single-centered study, so there will be a generalizability issue.

## Conclusions

The sequential use of sets of techniques was observed to be associated with improved outcomes in terms of blood loss, wound infection, mobility, patient satisfaction, and leading to the ultimate achievement of a dry Aquacel wound dressing that was not disturbed for a period of two weeks. Technically, the wound ooze can be significantly reduced despite the use of potent anticoagulants. Also, regardless of the use of potent anticoagulants, no patient developed deep vein thrombosis in this series.
